# Complete genome sequence of *Thioclava* strain BDS19, isolated from coastal marsh sediment

**DOI:** 10.1128/mra.00855-25

**Published:** 2025-11-26

**Authors:** Biagio DiSalvo, Blair G. Paul, Zoe G. Cardon

**Affiliations:** 1 The Ecosystems Center, Marine Biological Laboratory 42700, Woods Hole, Massachusetts, USA; 2 Bay Paul Center for Comparative Molecular Biology and Evolution, Marine Biological Laboratory 42700, Woods Hole, Massachusetts, USA; California State University San Marcos, San Marcos, California, USA

**Keywords:** *Thioclava*, genomes, sulfur oxidation, sulfur oxidizer, salt marsh

## Abstract

Salt marsh sediments host intensive sulfur cycling. Genome sequences from culturable sulfur-oxidizing microbes are required for targeted engineering to test *sox* gene function. We report the complete genome sequence (3,410,538 bp, 59.6 mol% G + C content) of putative sulfur oxidizer *Thioclava* strain BDS19, isolated from marsh sediment on Cape Cod, USA.

## ANNOUNCEMENT

Sulfur-cycling microbes are integral members of coastal marsh ecosystems (1). Heterotrophic sulfate reducers transform ocean-derived sulfates into energy-containing forms such as sulfide and thiosulfate, which sulfur oxidizers can then use to fuel growth. The forms of oxidized and reduced sulfur present in marsh sediments are important, as, for example, sulfide can be toxic to aerobic respiration in diverse plants (2). Though microbes involved in linked sulfur- and carbon-cycling have been studied (3, 4), genetic tools are needed to characterize the functionality of genes underlying these processes. Complete genomes provide necessary maps to identify target genes of interest and enable the design of sequence-specific engineering tools (5).

An isolate of *Thioclava* was obtained from pink sediment mats collected in March 2025 at *Sporobolus alterniflorus*-dominated Little Sippewissett Marsh [41°34′34.0″N, 70°38′18.8″W], Falmouth, MA. Bacteria in this genus are gram-negative, rod-shaped *Alphaproteobacteria* with ecological functions including carbon dioxide fixation, crude oil degradation, and sulfur oxidation (6, 7). They can be halotolerant, facultative anaerobes, and mixotrophic (8). Here, we report the complete genome sequence of *Thioclava* strain BDS19 as a first step toward engineering to understand regulation and function of sulfur cycling-related genes.

Briefly, a suspension of sediment was plated onto Luria-Bertani (LB) agar plates and incubated at 20–22°C for 1 week. Individual pale orange colonies were T-struck to obtain a pure culture. Strain BDS19 was stored in 25% (w/v) glycerol at −80°C. For whole genome sequencing, cells were harvested from 1-week-old plates, inoculated into LB broth, and incubated in a shaker incubator at 30°C at 193 rpm overnight. Genomic DNA was extracted using the NEB Monarch Genomic DNA Extraction and Purification Kit (#T3010S, NEB, USA).

Libraries were generated by Plasmidsaurus (Louisville, KY, USA) using an Ultra-long Sequencing Kit V14 by Oxford Nanopore Technology (ONT) (Lexington, MA, USA) and sequenced on PromethION R10.4.1 flow cells. Default parameters were used for all software unless otherwise specified. ONT’s Dorado (10.4.1) was used for basecalling and SPRI beads (Omega Bio-Tek, Georgia, USA) for size selection. A total of 267,568,203 bp with 74,581 reads were sequenced, and N50 was 9,100 bp. Filtlong (v0.2.1) (9) filtered out the lowest 5% of FASTQ reads by quality and downsampled the reads to 250 Mb to create a rough assembly using Miniasm (v0.3) (10). The information generated by Miniasm was used to further downsample to gather reads at up to ~100× coverage, with the parameter fitlong-mean_q_weight 10 applied to remove additional low-quality reads. The resulting subsample reads were used by Flye v2.9.1 (11) to call the circularity of the chromosome and generate a final assembly, which was further processed with Medaka v1.8.0 (12).

Total genome size was 3,410,538 bp with a G + C content of 59.6 mol%. Estimated completeness was 98.8% with 0.15% contamination (CheckM [v.1.2.2]) (13). Mash (v.2.3) (14) identified its closest related species as *Thioclava indica* (NZ_AUBN01000001.1) with 90.4% identity. Genome annotation was performed by NCBI Prokaryotic Genome Annotation Pipeline (v.6.10) (15) and identified 3,297 putative genes, 48 tRNAs, 9 rRNAs, and 4 ncRNAs. Numerous sulfur-related genes were annotated, including a sulfur-oxidizing cluster, *soxABCXYZ*.

## Data Availability

The draft genome has been submitted to GenBank, accession number CP195919, under BioProject PRJNA1285420 and the raw sequence file is available under SRA accession number SRP604649.
